# Supplementary material for: Afucosylated Plasmodium falciparum-specific IgG is induced by infection but not by subunit vaccination
Source: Nat Commun. 2021 Oct 5;12:5838. doi: 10.1038/s41467-021-26118-w (PMC8492741; doi:10.1038/s41467-021-26118-w)
Supplement: Supplementary file 1 — Supplementary information [file 41467_2021_26118_MOESM1_ESM.pdf]

Afucosylated *Plasmodium falciparum*-specific IgG is induced by infection but not by subunit vaccination

- Supplementary information -

Authors: Mads Delbo Larsen,[1§] Mary Lopez-Perez,[2§] Emmanuel Kakra Dickson,[3] Paulina Ampomah,[4] Nicaise Tuikue Ndam,[5] Jan Nouta,[6] Carolien A M Koeleman,[6] Agnes L Hipgrave Ederveen,[6] Benjamin Mordmüller,[7,8] Ali Salanti,[2] Morten Agertoug Nielsen,[2] Achille Massougbodji,[9] C. Ellen van der Schoot,[1] Michael F. Ofori,[3] Manfred Wuhrer,[6] Lars Hviid[2,10*#], and Gestur Vidarsson[1*#]

[§] These authors contributed equally
[*]These authors jointly supervised this work
[#]Correspondence: Gestur Vidarsson (g.vidarsson@sanquin.nl) or Lars Hviid (lhviid@sund.ku.dk)

This PDF file includes:

Supplementary figures S1-S6
Supplementary tables S1-S4

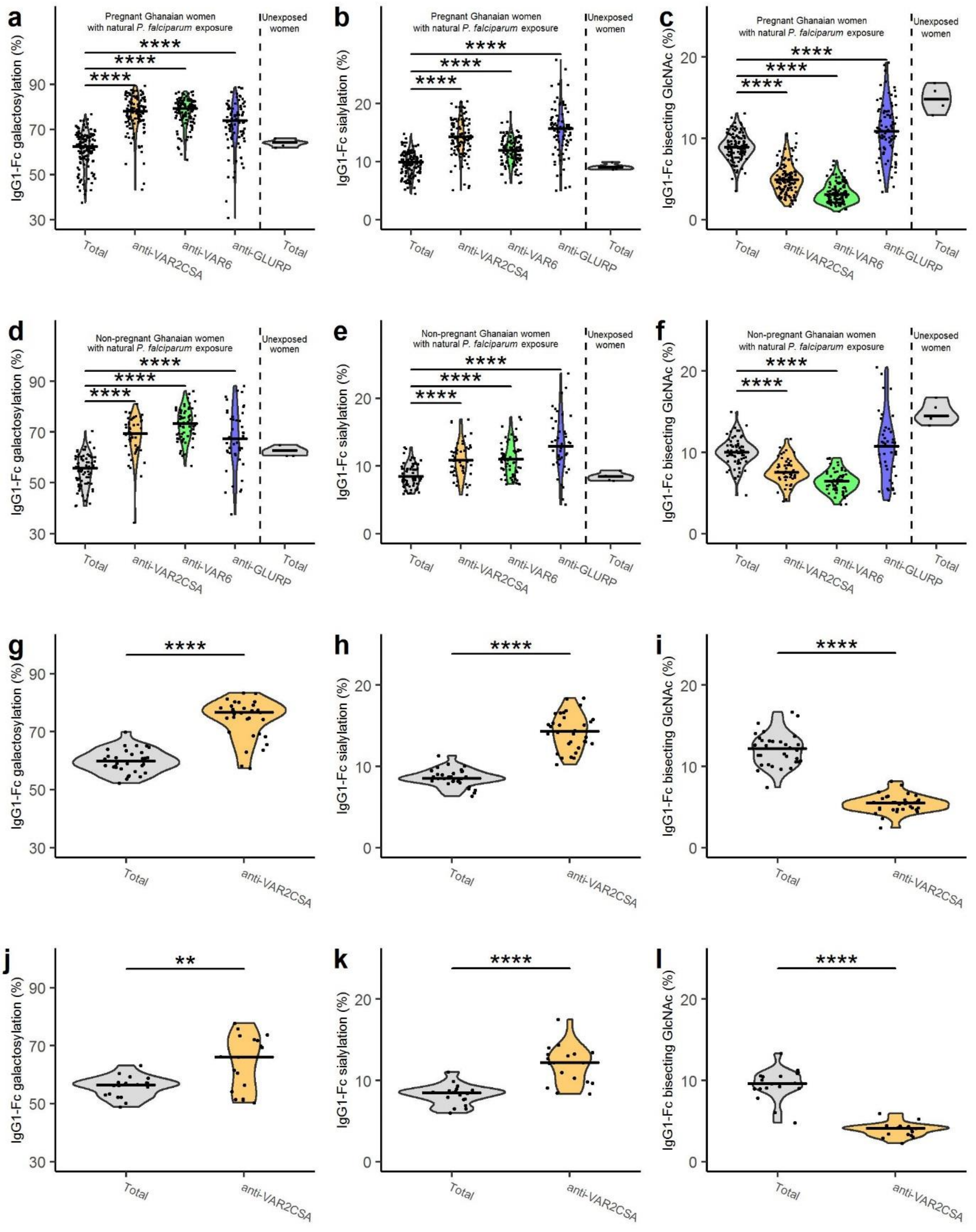

Supplementary Figure 1. Fc glycosylation traits of *P. falciparum*-specific IgG in pregnant women **(a, d, g, and j)** Fc galactosylation-, **(b, e, h, and k)** Fc sialylation-, and **(c, f, i, and j)** Fc bisecting GlcNAc levels of total IgG1 (gray) and IgG1 with specificity for VAR2CSA (orange), VAR6 (green), and GLURP (blue) in **(a to c)** pregnant Ghanaian women (Total IgG n=127; VAR2CSA n=117; VAR6 n=121; GLURP = 88), **(d to f)** non-pregnant Ghanaian women (Total IgG n=72; VAR2CSA n=50; VAR6 n=65; GLURP = 43), **(g to i)** *P. falciparum*-naïve German (n =32) and **(j to l)** VAR2CSA-naïve Beninese vaccinees (n = 18). Medians and densities are shown. Statistically significant pairwise differences between antigen-specific IgG and total IgG (multiple two-sided Wilcoxon signed rank tests with Bonferroni correction) are indicated (**: P<0.01; ****: P<0.0001)..

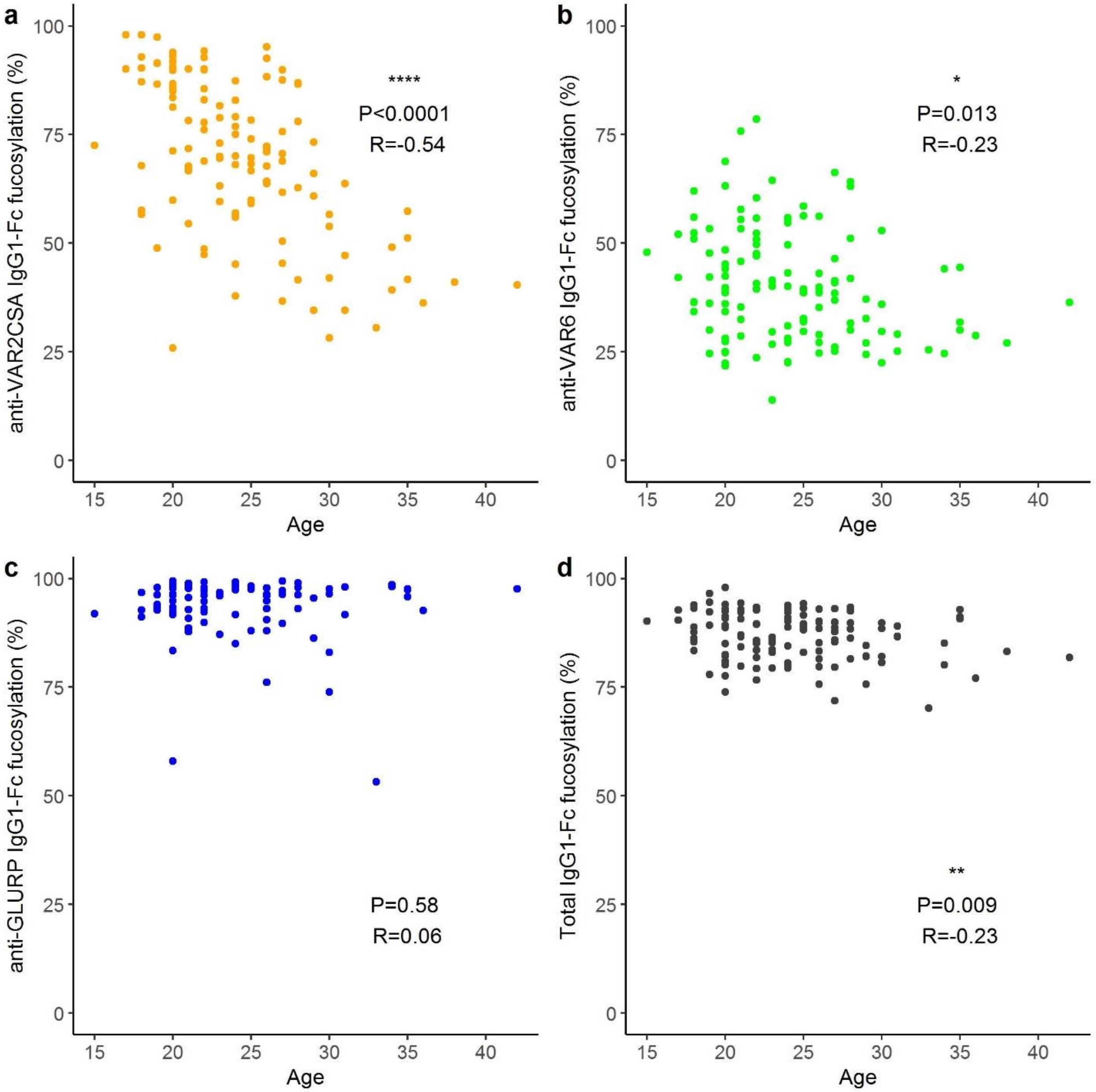

Supplementary Figure 2. Fc fucosylation of naturally acquired PfEMP1-specific IgG correlates with age

Correlations of **(a)** VAR2CSA- (n=117), **(b)** VAR6-(n=121), **(c)** GLURP-specific (n=88) and **(d)** total IgG1-Fc fucosylation levels (n=127) with age. P-values, and correlation coefficients are shown. Statistical significance of Spearman's correlations. *: P<0.05; **: P<0.01; ***: P<0.001; ****: P<0.0001.

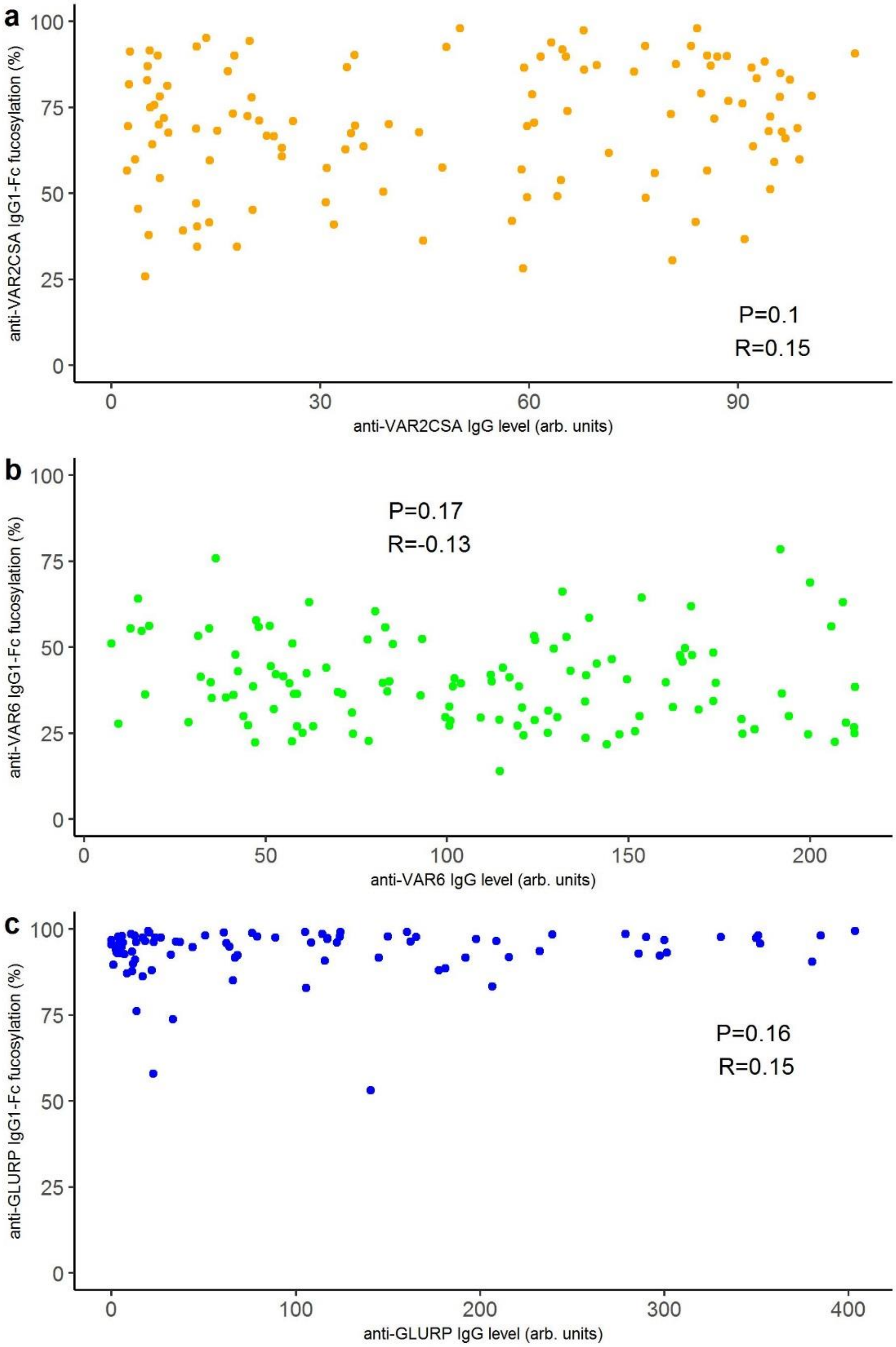

Supplementary Figure 3. No correlation between IgG levels and Fc fucosylation. Correlations of IgG levels and Fc fucosylation of **(a)** VAR2CSA-(n=117), **(b)** VAR6-(n=121), and **(c)** GLURP-specific (n=88) IgG1. Spearman's coefficient and P values are shown.

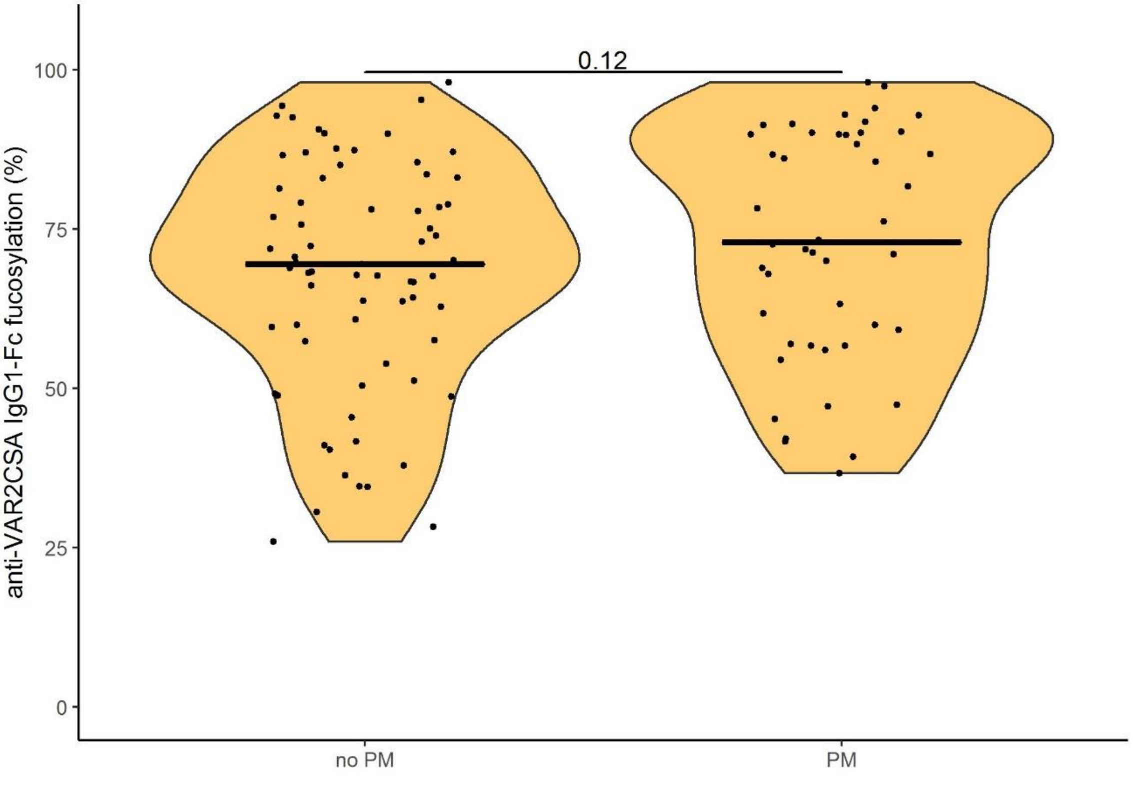

Supplementary Figure 4. No PM-dependent difference in Fc fucosylation levels of VAR2CSA-specific IgG. IgG Fc fucosylation levels of VAR2CSA-specific IgG are shown for women with and without PM. Statistical significance was tested using a two-sided Mann-Whitney test. Median, P value, and density are shown.

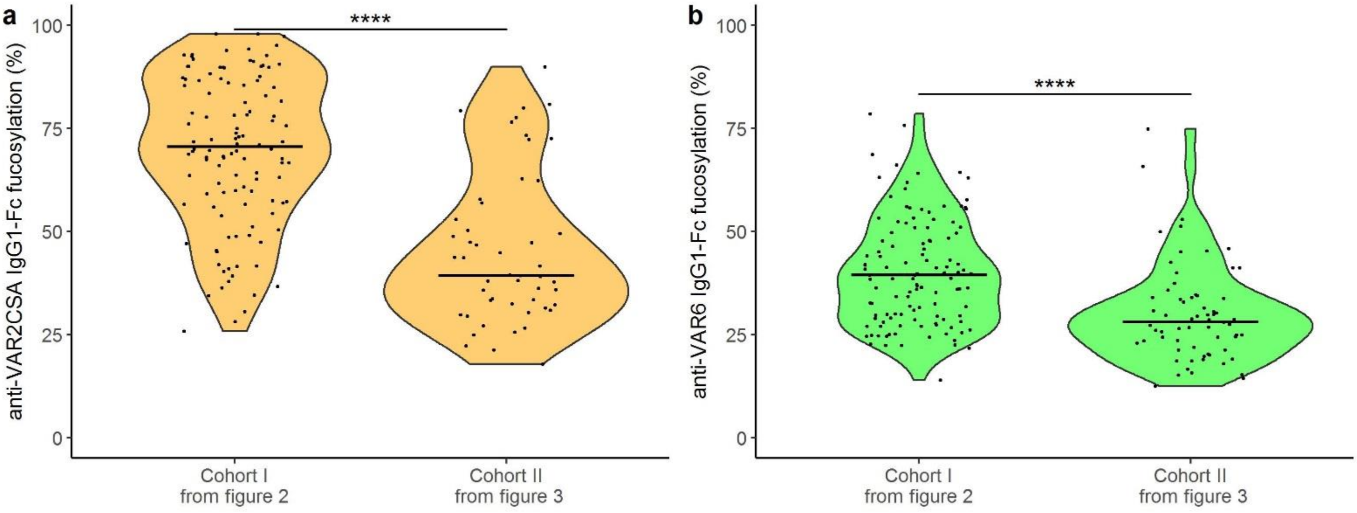

Supplementary Figure 5. Both VAR2CSA- and VAR6-specific Fc afucosylation are more pronounced in cohort of non-pregnant Ghanaian women. (a) VAR2CSA- and **(b)** VAR6-specific IgG Fc fucosylation of from the cohort of pregnant women and non-pregnant women. Data are aggregated from Figures 2-3. Statistically significant differences (Mann-Whitney) are indicated (****: P<0.0001).

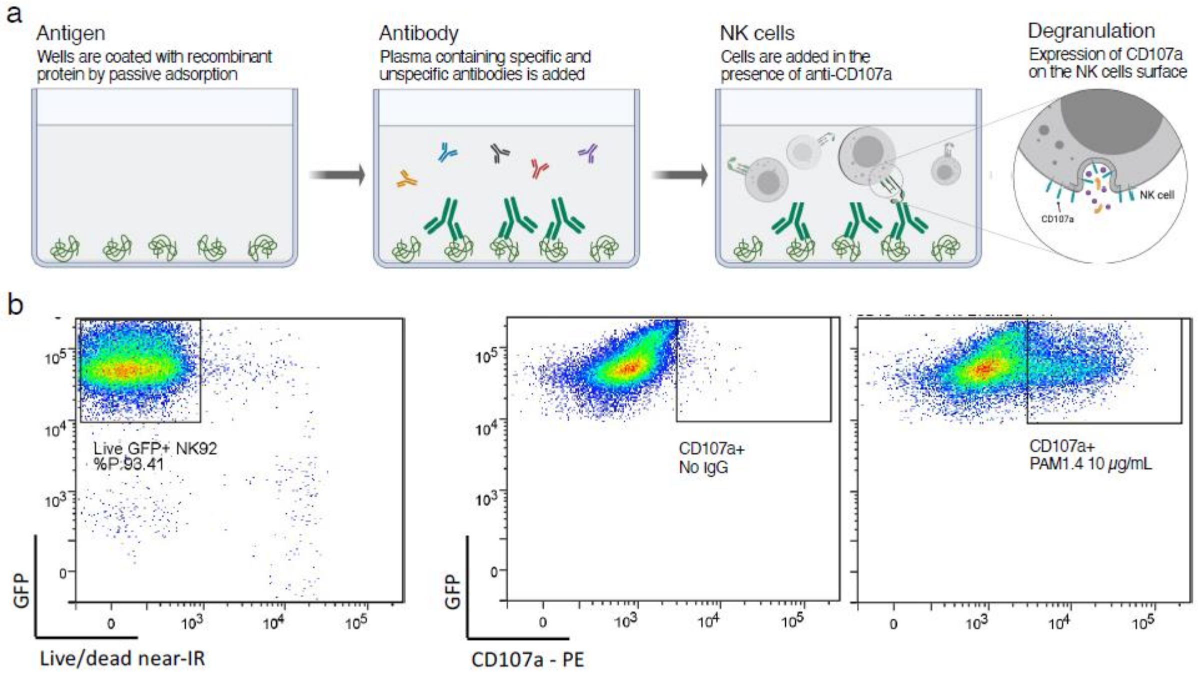

Supplementary Figure 6 . ADCC NK cell activation assay. (a) Schematic representation of plate-based assay. Created with Biorender.com. **(b)** Gating strategy for flow cytometry analysis. NK92 live cells expressing CD16 were gated as GFP$^+$ and live/dead$^-$. This population was used to estimate the percentage of NK cells expressing CD107a as a marker of ADCC. Representative dot plots of CD107a expression in the absence of antibodies (centre) or with 10 µg/mL human monoclonal anti-VAR2CSA (PAM1.4; right).

Supplementary Table 1

**Prediction of maternal hemoglobin level at birth by generalized linear model**			
**Variable**	**Coefficient**	**P value**	**Significance**
VAR2CSA IgG1 fucosylation (%)	-0.0173	0.0713	Not significant
Placental malaria	-0.583	0.0652	Not significant
Number of parities	-0.167	0.124	Not significant

P values and statically significance of each variable defined by a Wald-test of a generalized linear model including described variables are provided.

Supplementary Table 2

Prediction of birth weight by generalized linear model			
**Variable**	**Coefficient**	**P value**	**Significance**
VAR2CSA IgG1 fucosylation (%)	0.0038	0.187	Not significant
Placental malaria	-0.3271	0.0009	***
Number of parities	0.038	0.250	Not significant

P values and statically significance of each variable defined by a Wald-test of a generalized linear model including described variables are provided. (***: P<0.001)

Supplementary Table 3

Summary statistics of plasma donors studies

Cohort	Origin	Donors (N)	Women (N; %)	Age (median; inter-quartile range (in years))	Ref.
P. falciparum-exposed and pregnant	Ghana	127	127; 100%	24; 20-27	48
P. falciparum-exposed and non-pregnant women	Ghana	72	72; 100%	29; 23-38	53
Non-exposed vaccinees	Germany	36	n.a.[1]	Adults[1]	60
P. falciparum-exposed vaccinees	Benin	21	21; 100%	Adults[1]	(unpublished)

[1] Data not available due to blinding of the clinical trial data

Supplementary Table 4

Overview of included Fc glycopeptides			
N-Glycopeptide	m/z 2+	m/z 3+	Retention time (sec)
IgG1 H3N4F1S0 [G0F]	1317.526	878.687	80
IgG1 H4N4F1S0 [G1F]	1398.552	932.704	78
IgG1 H5N4F1S0 [G2F]	1479.579	986.722	77
IgG1 H3N5F1S0 [G0FN]	1419.066	946.380	81
IgG1 H4N5F1S0 [G1FN]	1500.092	1000.398	79
IgG1 H5N5F1S0 [G2FN]	1581.119	1054.415	78
IgG1 H3N4F0S0 [G0]	1244.497	830.001	83
IgG1 H4N4F0S0 [G1]	1325.524	884.018	82
IgG1 H5N4F0S0 [G2]	1406.550	938.036	81
IgG1 H3N5F0S0 [G0N]	1346.037	897.694	83
IgG1 H4N5F0S0 [G1N]	1427.063	951.712	82
IgG1 H5N5F0S0 [G2N]	1508.090	1005.729	79
IgG1 H4N4F1S1 [G1FS]	1544.100	1029.736	77
IgG1 H5N4F1S1 [G2FS]	1625.127	1083.754	75
IgG1 H4N5F1S1 [G1FNS]	1645.640	1097.429	77
IgG1 H5N5F1S1 [G2FNS]	1726.667	1151.447	77
IgG1 H4N4F0S1 [G1S]	1471.071	981.050	80
IgG1 H5N4F0S1 [G2S]	1552.098	1035.068	79
IgG1 H4N5F0S1 [G1NS]	1572.611	1048.743	77
IgG1 H5N5F0S1 [G2NS]	1653.638	1102.7610	77
IgG1 H5N4F1S2 [G2FS2]	1770.675	1180.786	76
